# A novel prognostic model of methylation-associated genes in acute myeloid leukemia

**DOI:** 10.1007/s12094-022-03069-2

**Published:** 2023-01-30

**Authors:** Meiyu Chen, Zhao Zeng, Wei Qin, Xiaohui Cai, Xuzhang Lu, Suning Chen

**Affiliations:** 1 Department of Hematology, The First Affiliated Hospital of Soochow University, Suzhou, 215006 Jiangsu China; 2 Department of Hematology, Affiliated Changzhou Second Hospital of Nanjing Medical University, Changzhou, 213003 Jiangsu China

**Keywords:** Methylation, Acute myeloid leukemia, Prognostic model, Risk score, Predictive

## Abstract

**Background:**

There is growing evidence that methylation-associated genes (MAGs) play an important role in the prognosis of acute myeloid leukemia (AML) patients. Thus, the aim of this research was to investigate the impact of MAGs in predicting the outcomes of AML patients.

**Methods:**

The expression profile and clinical information of patients were downloaded from public databases. A novel prognostic model based on 7 MAGs was established in the TCGA training cohort and validated in the GSE71014 dataset. To validate the clinical implications, the correlation between MAGs signature and drug sensitivity was further investigated.

**Results:**

76 genes were screened out by the univariate Cox regression and significantly enriched in multiple methylation-related pathways. After filtering variables using LASSO regression analysis, 7 MAGs were introduced to construct the predictive model. The survival analysis showed overall survival of patients with the high-risk score was considerably poorer than that with the low-risk score in both the training and validating cohorts (*p* < 0.01). Furthermore, the risk score system as a prognostic factor also worked in the intermediate-risk patients based on ELN-2017 classification. Importantly, the risk score was demonstrated to be an independent prognostic factor for AML in the univariate and multivariate Cox regression analysis. Interestingly, GSEA analysis revealed that multiple metabolism-related pathways were significantly enriched in the high-risk group. Drug sensitivity analysis showed there was a significant difference in sensitivity of some drugs between the two groups.

**Conclusion:**

We developed a robust and accurate prognostic model with 7 MAGs. Our findings might provide a reference for the clinical prognosis and management of AML.

**Supplementary Information:**

The online version contains supplementary material available at 10.1007/s12094-022-03069-2.

## Background

Acute myeloid leukemia (AML) is a common hematological malignancy, featured by abnormal proliferation and blocked differentiation of hematopoietic stem cells [1]. Abnormality in cytogenetics and molecular biology is intimately related to the response of leukemia cells to chemotherapy and the prognosis of AML patients. Although there are two major leukemia therapies including chemotherapy and hematopoietic stem cell transplantation, the addition of some molecularly targeted drugs has significantly improved the response rate to chemotherapy. Nevertheless, the majority of patients achieved a short remission and eventually suffered disease progression and relapse [2]. Furthermore, there is a larger heterogeneity among AML patients including clinical manifestations and disease prognosis. With the development of next-generation sequencing and transcriptome sequencing, the heterogeneity has been discovered and becomes more evident at the genetic level. The gene expression profile is closely associated with cytogenetic or molecular abnormalities. For example, over-expression of the BAALC gene was considered as an independent risk factor, which was usually accompanied by FLT3-ITD and MLL-PTD and associated with poor overall survival (OS) [3]. Establishment of the 2017 European Leukemia-Net (ELN) risk stratification system was based on previous research on cytogenetic and molecular abnormalities, but it did not incorporate the gene expression profiles which played an important role in disease development. Therefore, accurate and systematic prognostic assessment before treatment is particularly important for patients.

A series of changes in the structure and function of the genome were associated with the occurrence and development of tumors, which featured with transcriptional dysregulation and abnormal gene expression [4]. Furthermore, genomic abnormalities result in changes in epigenetic modification, which have a profound impact on the stabilization of chromatin structure. Epigenetic modifications play an important role in tumorigenesis, progression, and metastasis [5]. It has been shown that at least four epigenetic events were related to gene expression alterations, including DNA methylation, post-translational modifications of histones, chromatin remodeling, and RNA-mediated mechanisms [6]. It was reported that the methylation signature of the majority of HOX genes was common anomalous in a wide variety of tumors and was considered as an important diagnostic and prognostic biomarker [7]. A delicate balance between histone methylation and demethylation was also associated with the development and physiological function of the normal cell. The aberrant methylation of histone was identified as a possible biomarker and potential therapeutic target in pancreatic cancer patients [8].

As discussed previously, AML is frequently accompanied by cytogenetic abnormalities and aberrant gene expression patterns, as well as alterations in epigenetics and proteomics. The above-mentioned alterations are related to the pathogenesis of leukemia and are determinants of disease progression and clinical prognosis. As we all know, the ELN 2017 risk stratification is considered as the classic criterion for prognosis and has been widely used. However, the traditional standard could not adequately predict the outcome of all AML patients. More comprehensive and reliable prognostic classification is urgently needed. Consequently, we utilized the available data from the public database and established a prognostic model of AML in the TCGA cohort based on MAGs, which was then verified in the GSE71014 cohort. In conclusion, MAGs signature was generated to predict the outcome of AML and provided more accurate stratification management of AML.

## Materials and methods

### Data download and processing

The expression profile and clinical information of TCGA-LAML samples were obtained from the UCSC Xena database (http://xena.ucsc.edu/). We further downloaded the transcriptomic data of normal human peripheral blood as a control from GTEx database. We normalized the raw counts and filtered out extremely low-expressed genes by the DESeq2 R package. The GSE71014 Series Matrix File data were obtained from the Gene Expression Omnibus (GEO) database. The annotation platform was GPL10588. The inclusion criteria of all specimens in the training and validating cohorts were as follows: (1) de novo acute myeloid leukemia excluding M3; (2) survival information corresponding to individual samples (3) the transcriptomic data corresponding to individual samples.

### Gene function and pathway analysis of MAGs

The GeneCards database was searched for methylation-associated genes (MAGs). (https://www.genecards.org/). “Methylation” was set as a search term in the database. The filtering criteria were described as follows: (1) Category was protein-coding; (2) Relevance score was greater than 5. We performed the univariate Cox regression analysis to screen for MAGs whose expression levels were associated with survival. We comprehensively explored the function of the prognostic MAGs using Gene Ontology (GO) and Kyoto Encyclopedia of Genes and Genomes Functions (KEGG) Enrichment Analysis. ClusterProfiler R package was utilized [9]. The gene network constructed by STRING v11.5 [10] was viewed and analyzed with Cytoscape [11]. Furthermore, Cytoscape Network Analyzer [11] was performed to calculate the degree distribution.

### Establishment and evaluation of prognostic model

TCGA cohort was defined as a training set for the establishment of a predictive model. The prognostic MAGs were identified by univariate Cox regression analysis in the training set. We performed the univariate Cox regression analysis of 362 MAGs using the ‘survival’ package in R software to obtain the prognostic genes (*p* < 0.05). Further analysis to filter variables was performed by the least absolute shrinkage and selection operator (Lasso) regression analysis, which utilized Lasso regularization with 1000-fold cross-validation to prevent overfitting [12]. The optimal values for the penalty parameter *λ* were set based on minimal criteria. The formula for risk score was based on variables with non-zero coefficients. The risk score of each patient was generated according to the unified algorithm. Patients were divided into high- and low-risk groups based on the best cut-off value of risk score. The GSE71014 dataset was defined as a validation set to evaluate the accuracy and specificity of the risk model. We utilized the log-rank test to compare differences in survival between high- and low-risk groups. We plotted survival curves by the Kaplan–Meier method. The time-dependent receiver operating characteristic (ROC) curve analysis was performed to estimate the accuracy of the risk model.

### Gene set enrichment analysis

To compare the differences in expression profiles between the high- and low-risk groups in the training cohort, we utilized GSEA software using the c2.cp.kegg.v7.0.symbols gene sets to identify potential biological pathways [13].

### Drug sensitivity analysis

We obtained the gene expression profiles and drug-response data of different tumor cell lines from The Genomics of Drug Sensitivity in Cancer (GDSC) database. We utilized the “oncoPredict” R package to predict the response of each patient in the TCGA cohort to chemotherapy drugs. The estimated value of the half-maximum inhibitory concentration (IC50) of each compound was calculated by Ridge’s regression. Tenfold cross-validation was performed in the GDSC training set to assess the accuracy of the drug-response prediction [14].

### Statistical analysis

In both the training and validating cohorts, the calculation of the area under the curve (AUC) was performed to evaluate the predictive performance of methylation-associated genes signature using the survival ROC package. Both univariable and multivariable Cox analyses were utilized to assess the predictive performance of clinical and genetic characteristics. The variables that were significant in the univariate analysis were included in the multivariate Cox analysis. A significant difference was indicated by *p* < 0.05 for all statistical tests.

## Results

### Biological function analysis of MAGs

By searching the GeneCards database with “methylation” as a search term and filtering for the above criteria, we obtained a gene set with 362 MAGs (Supplementary Table 1). We combined the expression profile with survival information and identified 76 genes with potential prognostic values by univariate Cox regression (Supplementary Table 2). GO enrichment analysis was conducted to investigate the biological functions of 76 prognostic MAGs. Supplementary Fig. 1A depicted the top 10 terms for each GO category, including biological process (BP), cellular component (CC), and molecular function (MF). In biological processes, 76 MAGs were mainly enriched in methylation, macromolecule methylation, protein methylation, and protein alkylation. In the cell component, 76 MAGs were mainly enriched in methyltransferase complex, ESC/E(Z) complex, histone methyltransferase complex, and nuclear heterochromatin. Regarding molecular function, 76 MAGs were mainly enriched in methyltransferase activity, S-adenosylmethionine-dependent methyltransferase activity, transferase activity, and protein methyltransferase activity (Supplementary Table 3). Moreover, the result of the KEGG enrichment analysis was shown in Supplementary Fig. 1B. The enriched KEGG pathways were associated with colorectal cancer, FOXO signaling pathway, microRNAs in cancer, and lysine degradation (Supplementary Table 4). To identify the interactions among the 76 genes, we utilized the STRING online database. Furthermore, the cluster of 10 highly interconnected genes was revealed by Cytoscape plug-in MCODE (Supplementary Fig. 1C).

### Establishment and evaluation of prognostic model

**Fig. 1:**
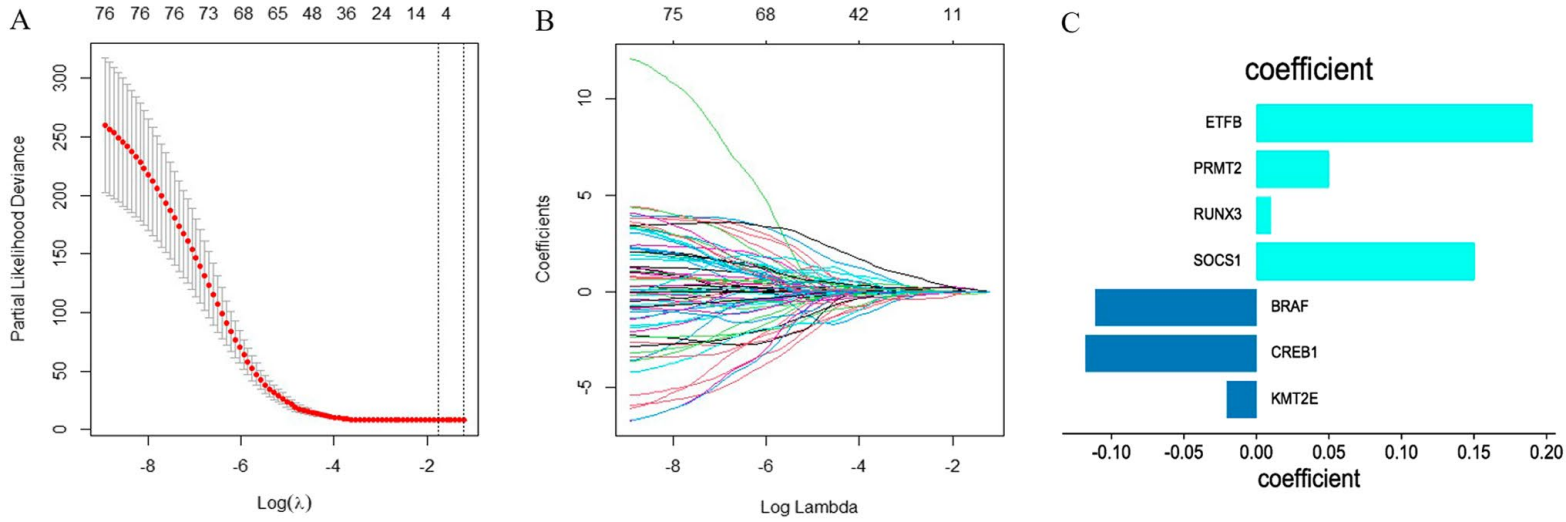
Construction of MAG-based predictive model. **A** 1000-fold cross-validation to select variables by the LASSO regression with the optimal *λ* value (**B**) LASSO coefficients of methylation-associated genes. Each curve represents a methylation-associated gene. **C** The non-zero regression coefficient of 7 prognostic MAGs

**Fig. 2:**
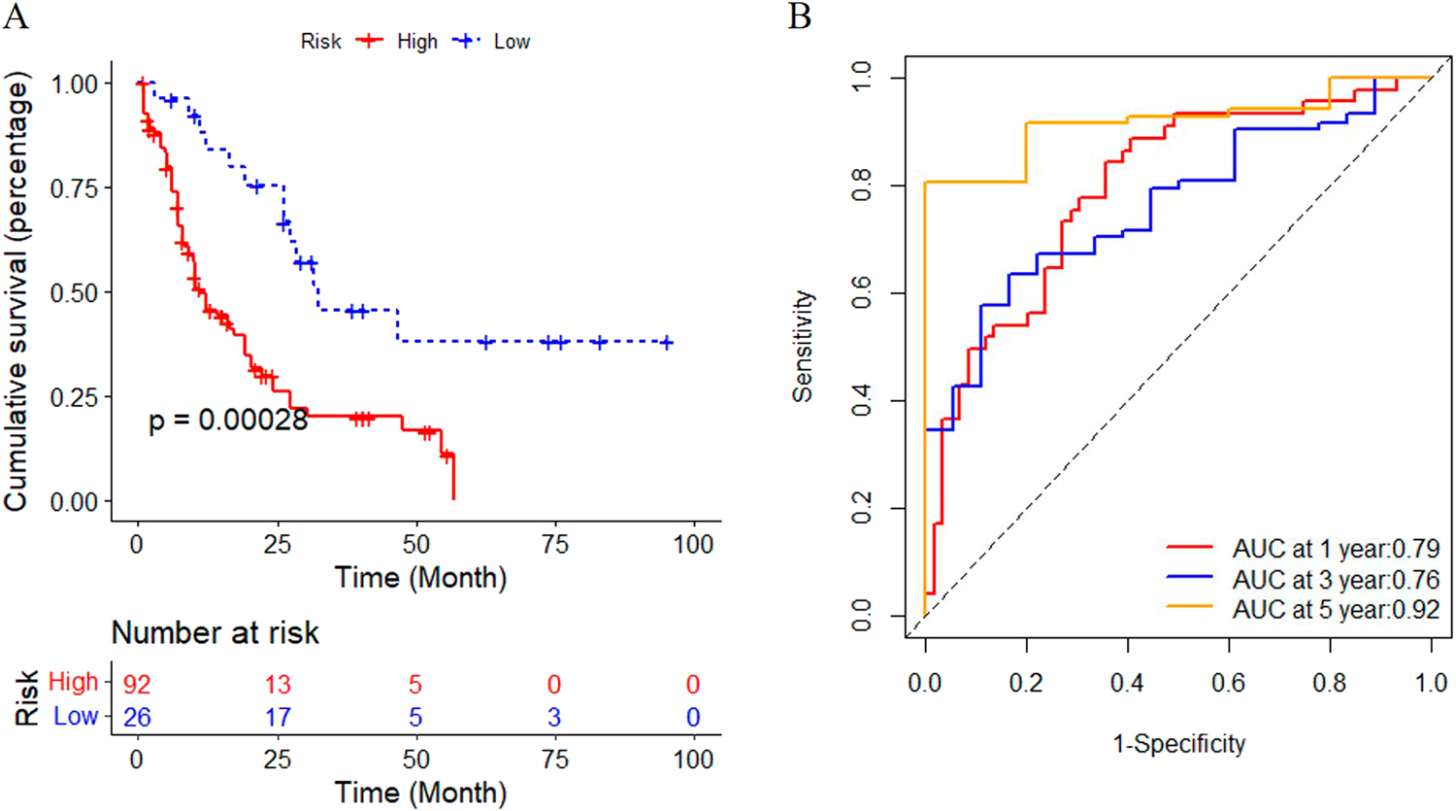
**A** Assessment of survival differences between the high- and low-risk groups in the training cohort, in which 92 samples were assigned into the high-risk group and 26 samples into low-risk group, respectively. The red line represents the high-risk group and the blue line represents the low-risk group. **B** ROC analysis of risk model in the training cohort. AUC values of 1, 3, and 5 years are 0.79, 0.76, 0.92

**Fig. 3:**
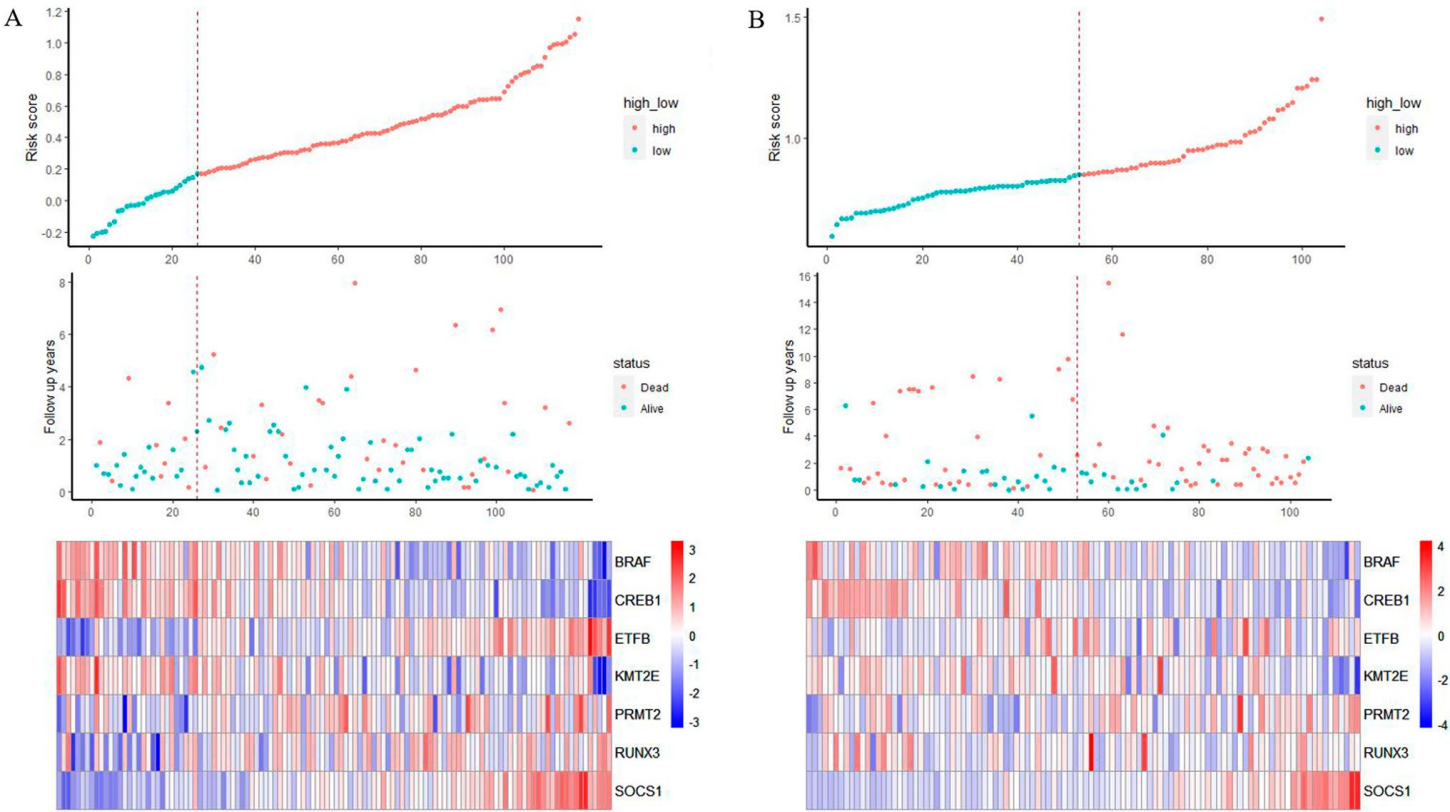
**A** The risk scores distribution, survival status and the expressions of 7 MAGs in the training cohort. **B** The risk scores distribution, survival status and the expressions of 7 MAGs in the validating cohort

Lasso regression analysis identified 7 genes with a non-zero regression coefficient (Fig. 1). The risk score was calculated based on the following algorithm: risk score = BRAF * (− 0.11119721) + CREB1 * (− 0.1179764) + KMT2E * (0.02048181) + ETFB * (0.1905194) + PRMT2 * (0.04986465) + RUNX3 * (0.01006469) + SOCS1 * (0.15040067). On the basis of the best risk cut-off value, patients were classified into high- and low-risk groups. Additionally, the survival analysis revealed that OS of the high-risk patients was considerably shorter than that of the low-risk patients in the TCGA cohort (*p* = 0.00028, Fig. 2A). As shown in Fig. 2B, the AUC values for 1-, 3-, and 5-year OS were 0.79, 0.76, and 0.92, respectively, demonstrating excellent predictability for the prognosis-associated risk model. The correlation of risk score, survival status, and the expression of the 7 MAGs was described in Fig. 3A–C. We discovered that the expressions of genes with hazard ratios > 1, such as ETFB, PRMT2, RUNX3, and SOCS1, were expressed more in high-risk patients, whereas the expressions of genes with hazard ratios < 1, such as BRAF, CREB1, and KMT2E, were expressed more in low-risk patients (Supplementary Fig. 2).

**Fig. 4:**
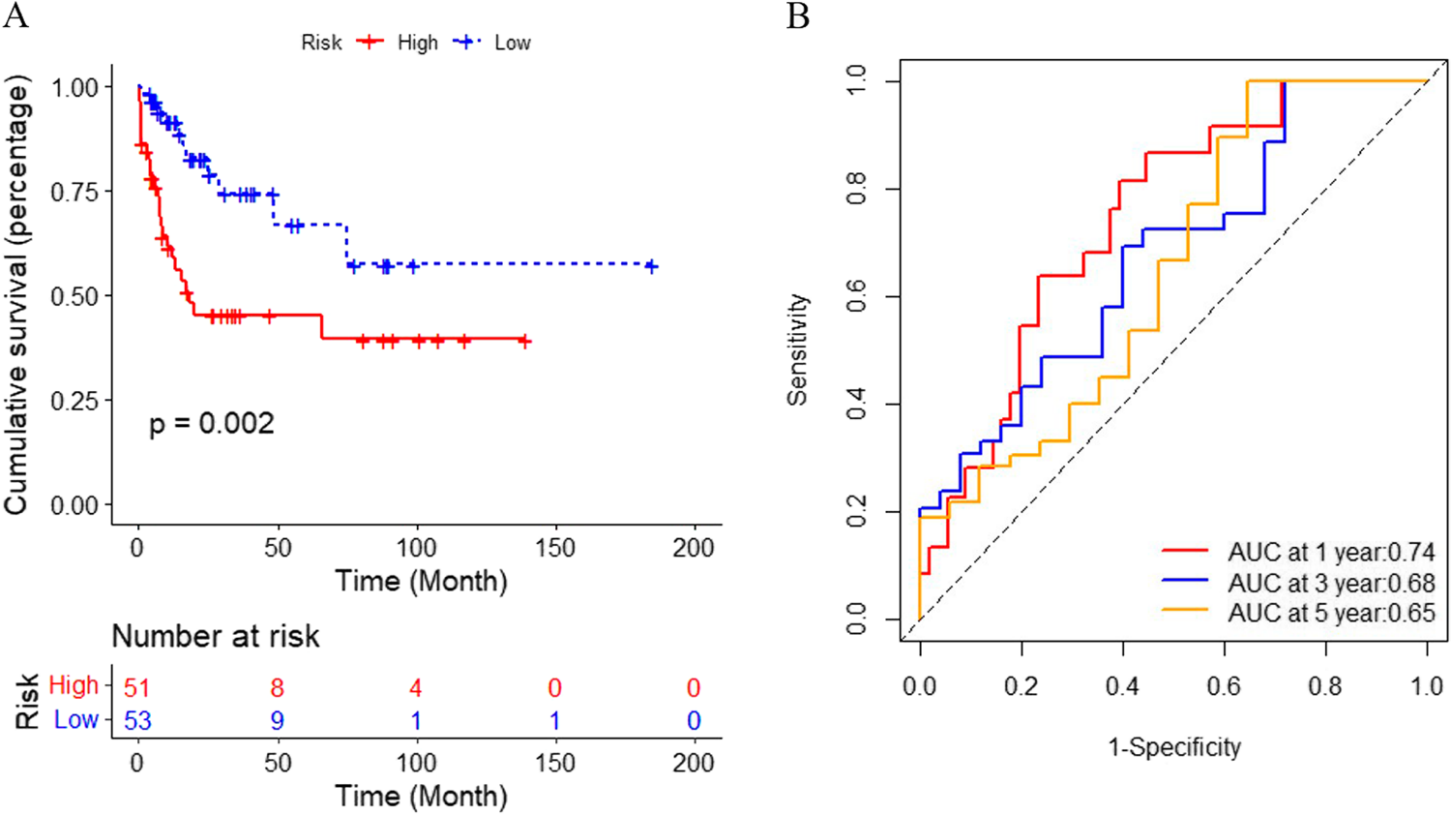
**A** Assessment of survival differences between the high- and low-risk groups in the validating cohort, in which 51 samples were assigned into the high-risk group and 53 samples into low-risk group, respectively. The red line represents the high-risk group and the blue line represents the low-risk group. **B** ROC analysis of risk model in the validating cohort. AUC values of 1, 3, and 5 years are 0.74, 0.68, 0.65

Next, the GSE71014 dataset was described as a validation cohort to assess predictive performance. We discovered similar results based on the unified formula, demonstrating the robustness of the risk model. High-risk patients had significantly shorter overall survival than low-risk patients (*p* = 0.002, Fig. 4A). Moreover, the AUCs for 1-, 3-, and 5-year OS were 0.74, 0.68, and 0.65, respectively (Fig. 4B). The correlation of risk score, survival status, and the expression profile of the validation cohort were shown in Fig. 3D–F. The results fully demonstrated the robustness and accuracy of the risk model.

### Independent prognostic significance of risk score

The univariate and multivariate Cox regression analyses were conducted on the training dataset to assess the independence of MAGs signatures in clinical applications. The risk scores and clinical characteristics, including gender, age, white blood cell count, bone marrow blast cell, DNMT3A mutation, IDH1 mutation, IDH2 mutation, TET2 mutation, and risk cytogenetic, were used as covariates. According to the univariate Cox analysis, age, DNMT3A mutations, risk score, and risk cytogenetic were significantly associated with survival. Further multivariate analysis revealed that age, DNMT3A mutation, and risk score were independent predictive factors of survival, and risk score was superior to age and DNMT3A mutation (Table 1).

**Table 1:** The univariate and multivariate Cox analysis of clinical characteristics and risk score in TCGA cohort

Variables	Univariate Cox			Multivariate Cox		
	Hazard ratio	95% CI	*p* value	Hazard ratio	95% CI	*p* value
Gender	1.08	0.68–1.71	0.74	NA	NA	NA
Age	1.03	1.01–1.04	**9.1E−04**	1.02	1.00–1.04	**1.79E−02**
WBC	1.00	1.00–1.01	0.28	NA	NA	NA
BM blast	1.00	0.99–1.01	0.78	NA	NA	NA
DNMT3A mutation	2.11	1.27–3.51	**3.8 E−03**	1.73	1.02–2.92	**4.14E−02**
IDH1 mutation	0.77	0.37–1.60	0.48	NA	NA	NA
IDH2 mutation	0.90	0.45–1.81	0.77	NA	NA	NA
TET2 mutation	1.28	0.64–2.57	0.49	NA	NA	NA
Risk_cytogenetic	1.55	1.07–2.25	**0.02**	1.38	0.89–2.15	1.46E−01
Risk score	11.75	5.18–26.65	**3.64E−09**	7.96	3.32–19.07	**3.29E−06**

*WBC* white blood cell, *BM* bone marrow

**Fig. 5:**
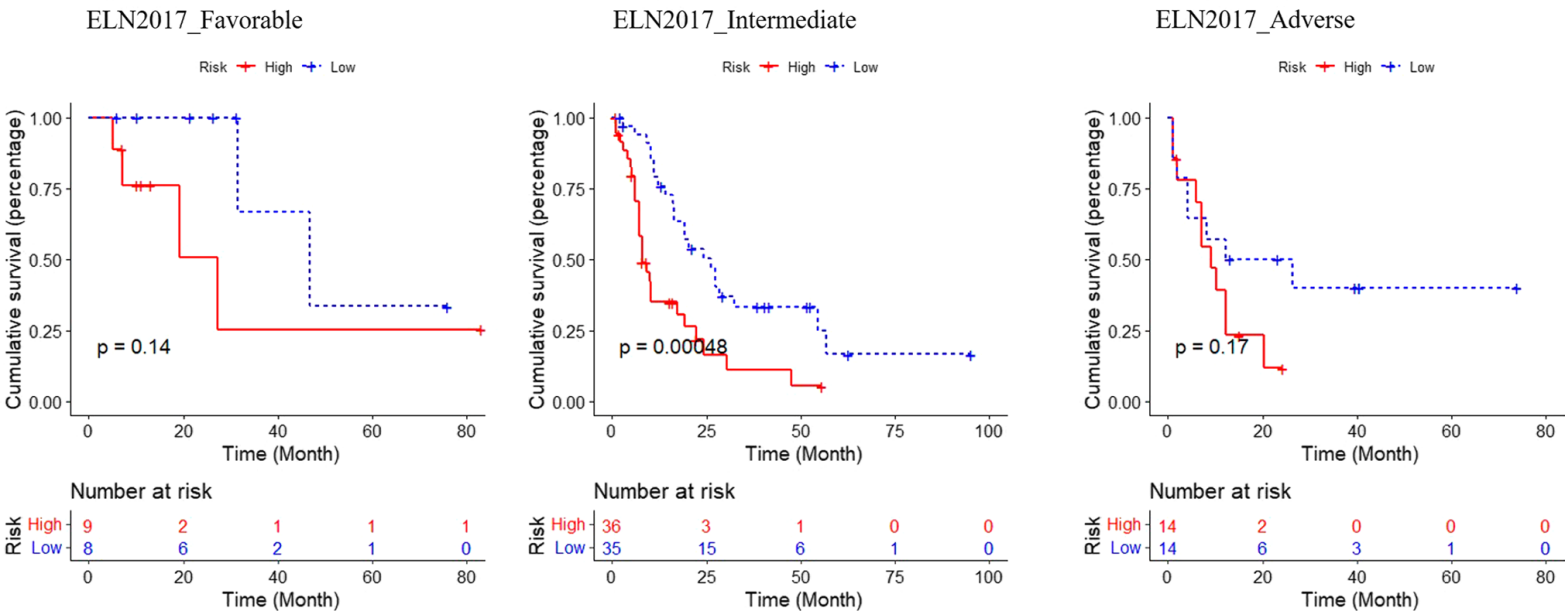
The analysis of risk score in combination with ELN 2017 risk stratification. Patients were divided into the three ELN 2017 categories. Kaplan–Meier analysis was used for these patients re-stratified by risk score

Following that, we investigated the clinical utility of incorporating the risk score into the ELN 2017 risk stratification. The results indicated that the risk score could discern low-risk patients from high-risk patients in ELN 2017 intermediate group (Fig. 5). However, risk scores did not distinguish the patients in favorable and adverse groups, possibly due to the small number of patients in these two groups. We believed that risk score in combination with ELN 2017 risk stratification could more accurately define the prognosis of AML patients.

### The specific signaling pathways related to the predictive model

**Fig. 6:**
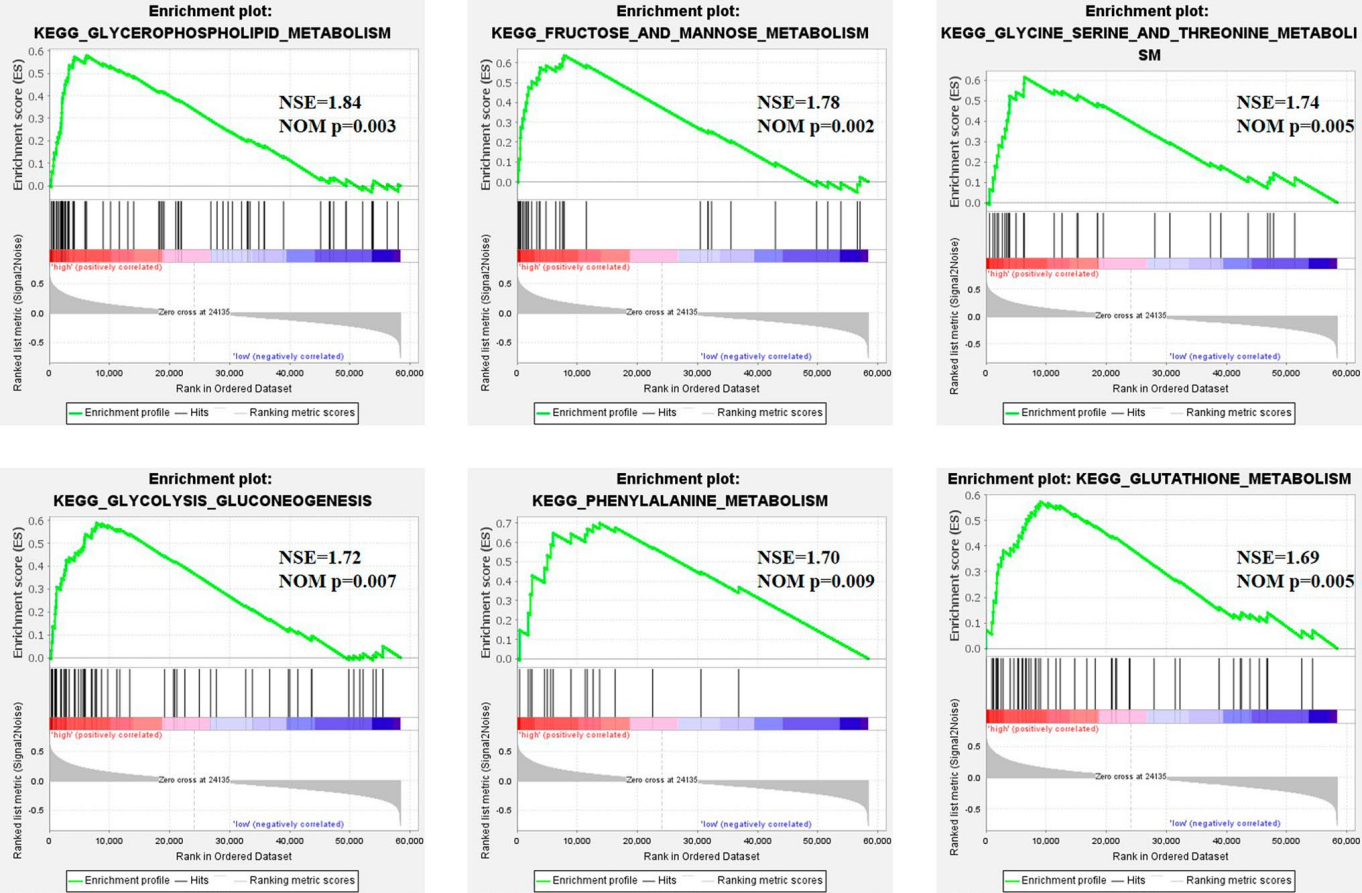
The significant enrichment of some pathways in KEGG analysis. Patients in the high-risk group were mainly enriched in metabolism-related signaling pathways

GSEA was performed in the TCGA cohort to investigate the specific pathways connected to the predictive model. The high-risk patients had significantly enriched pathways, the majority of which were metabolism-related (Supplementary Fig. 3A). These pathways included glycero-phospholipid metabolism, fructose and mannose metabolism, glycine serine and threonine metabolism, glycolysis glyco-isomerism, phenylalanine metabolism, and glutathione metabolism (Fig. 6). Besides, as shown in Supplementary Fig. 3b, 6 pathways were highly enriched in low-risk patients, which included ascorbic acid and aldehyde metabolism. Therefore, the above results suggested that risk stratification based on MAGs was highly associated with metabolic abnormalities.

### Different responses to drugs between high‑ and low-risk groups

We utilized the “oncoPredict” R package to predict the responses to 198 compounds of each patient. Drug sensitivity analysis revealed significant differences in 55 chemotherapy drugs between high- and low-risk patients, which might contribute to personalized treatment for AML (Supplementary Table 5). As shown in Supplementary Fig. 4, the top ten drugs with the most significant *p* values were presented. Generally, PF-4708671, Pictilisib, and AZD2014 were more suitable for high-risk patients. Low-risk patients might benefit from the remaining seven drugs, including UMI-77, RO-3306, and IAP-5620.

## Discussion

In the current study, a novel predictive model of AML based on 7 MAGs was established and validated. Patients can be categorized into the high-risk or low-risk group using the model. Furthermore, the model worked well and accurately predicted the OS of AML patients both in the training and the validating cohort. The risk score was identified as the independent predictive factor according to the univariate and multivariate analyses. The risk score had a greater impact on the AML risk category and patients’ survival among all clinical parameters, which might effectively guide the prognosis of AML.

Four in seven MAGs of the risk model including ETFB, PRMT2, RUNX3, and SOCS1 were highly expressed in high-risk patients and were recognized as risk genes associated with poor prognosis, whereas the rest of 3 genes BRAF, CREB1, and KMT2E, were highly expressed in low-risk patients, indicating risk genes were associated with favorable prognosis. ETFB, as an electron transfer flavoprotein subunit beta, formed a complex with ETFA, one flavin adenine dinucleotide (FAD), and one adenosine monophosphate (AMP). By transporting electrons between flavoprotein dehydrogenases, they contributed to the metabolism of fatty and amino acids in the mitochondria [15]. Oxygen consumption rate in permeabilized mitochondria was influenced by ETFB methylation, suggesting that ETFB methylation could also regulate mitochondrial metabolism [16]. As a member of the protein arginine methyltransferase (PRMT) family, PRMT2 had been demonstrated to catalyze the arginine methylation of target proteins and link them to the development and progression of cancers [17]. RUNX3, as a transcription factor, belonged to the Runt-related transcription factor (RUNX) family. RUNX3 interacted with beta subunits to bind to DNA sequences, which activated or repressed transcription [18]. Previous studies reported that inactivation of the RUNX3 tumor-suppressor gene led to up-regulation of oncogenes expression and abnormalities in signaling pathways, which promoted the progression of various solid tumors [19]. Abnormal RUNX3 expression was associated with the sensitivity of leukemic cells to chemotherapeutic drugs [20]. Promoter methylation of the suppressor of cytokine signaling-1 (SOCS1) gene contributed to gene silencing, which was associated with the occurrence of malignant tumors. Zhang XH et al. reported that SOCS1 methylation affected JAK2/STAT signaling pathway and promoted the proliferation of leukemia cells [21]. BRAF acted as a part of the RAS/MAPK signaling pathway and had been implicated in regulating cell proliferation, differentiation, and apoptosis. The role of BRAF had been well documented in various solid tumors [22]. CREB1, as a cAMP response element binding protein-1, cooperated with AMPK and was involved in glioma cell proliferation. The study showed that persistent oncogenic stress activated the AMPK–CREB1 pathway and regulated the transcription levels of HIF1α and GABPA to support glioma bioenergetics [23]. KMT2E, also called MLL5, is incorporated into the lysine N-methyltransferase 2 (KMT2) family with other six SET methyltransferase domain proteins. And it is required for embryonic development as well as normal hematopoiesis [24]. Damm et al. reported the KMT2E transcript level represented an important factor influencing the survival of patients with normal karyotype. The study had demonstrated that KMT2E was an effective biomarker to predict sensitivity to decitabine [25].

The result of GSEA showed that the enriched pathways in high-risk AML patients were mainly connected to the metabolic pathways. High-risk patients showed a strong correlation to metabolic dysregulation, including glucose metabolism and amino acid metabolism. We consider metabolism and epigenetics to be involved in the development and progression of AML and emphasize the connection between the two cellular processes. Indeed, mounting evidence had pointed out that altered metabolic and epigenetic programs of AML were not completely independent from each other. It was hypothesized that the distinctive metabolic profile was crucial for the identification of AML cells since it could distinguish leukemia stem cells from normal hematopoietic stem cells [26]. Notably, this might have important implications for developing new therapeutic strategies. Recent studies had demonstrated that the Bcl-2 inhibitor Venetoclax which inhibited oxidative phosphorylation of LSCs in combination with DNMT inhibitor Azacytidine had a profound influence on the eradication of LSCs [27]. This synergistic effect indicated that epigenetics and metabolism were inextricably intertwined in AML. Therefore, it is worth further exploring the interesting relationship and the possibility of combination therapy of the two.

We utilized the GDSC database and explored the different responses to chemotherapy drugs in high- and low-risk patients. It showed that among chemotherapeutic drugs, such as PF-4708671, Pictilisib, and AZD2014, the IC50 in the high-risk group was lower, indicating that patients were more sensitive to these three drugs. PF-4708671 is a potent cell-permeable S6K1 inhibitor and few studies have reported the efficacy of PF-4708671 in AML. Recently, Spaan et al. reported a synergistic effect of PF-4708671 combined with MCL-1 inhibitor in inducing human myeloma cell lines apoptosis [28]. The PI3Kα inhibitor (Pictilisib) plus trametinib synergistically exerted anti-proliferative effects by increasing eEF2K activity [29]. The mTORC1/2 dual inhibitor AZD2014 demonstrated strong lysosome activation and increased the cytotoxic effect of Gemtuzumab ozogamicin, which was considered to be a suitable and clinically applicable agent for AML [30]. High-risk patients were less sensitive to UMI-77, RO-3306, and IAP-5620, whereas low-risk patients could benefit from them. Drug sensitivity prediction revealed a link between MAGs signature and prognosis in AML. Moreover, the analysis of drug sensitivity provided new directions in the treatment of AML.

Despite the robustness and accuracy of the risk model, there are some limitations. Due to the unavailable clinical information from the GEO database, we were unable to perform a specific analysis of the validation cohort. Moreover, the analysis reported in this study was a retrospective exploratory analysis based on public databases. Different preprocessing of the dataset and different clinical backgrounds of patients might result in biased prediction efficacy. Finally, the applicability of the risk model should be further evaluated in real-world research, and comprehensive experimental studies are required to clarify the exact molecular mechanism of these 7 genes in AML development, metabolic abnormalities, and drug resistance.

## Conclusion

In conclusion, a predictive model based on abnormal expression patterns of MAGs was constructed, which could be used as an independent predictive factor and had important clinical implications. Moreover, AML patients with high-risk scores were closely associated with metabolic pathway abnormalities, indicating that metabolic disorders were involved in the development of AML. This might be an important research direction and a novel therapeutic target. Additionally, the sensitivity of chemotherapy drugs had been assessed for MAGs risk signature. Our findings might provide a valuable reference for the clinical prognosis and personalized treatment strategies for AML.

## Supplementary Information

Below is the link to the electronic supplementary material.

Supplementary file1 Supplementary Fig. 1 The analysis of 76 prognostic MAGs. (A) The analysis of 76 prognostic MAGs based on GO enrichment analysis. (B) The analysis of 76 prognostic MAGs based on KEGG enrichment analysis. (C) Cluster analysis of interconnected genes by Cytoscape. (JPG 311 KB)

Supplementary file2 Supplementary Fig. 2 Forest plot of univariate Cox regression analysis of 7 MRGs for overall survival in the training cohort. (JPG 34 KB)

Supplementary file3 Supplementary Fig. 3 (A) Significantly enriched pathways in the high-risk group in the training cohort. (B) Significantly enriched pathways in the low-risk group in the training cohort. (JPG 186 KB)

Supplementary file4 Supplementary Fig. 4 The estimated IC50 of the top ten drugs with the most significant p values. Blue boxplots: IC50 values for the high-risk group; yellow boxplots: IC50 values for the low-risk group. (JPG 262 KB)

Supplementary file5 (JPG 99 KB)

Supplementary file6 (XLSX 41 KB)

Supplementary file7 (XLSX 16 KB)

Supplementary file8 (XLSX 11 KB)

Supplementary file9 (XLSX 10 KB)

Supplementary file10 (XLSX 10 KB)

Supplementary file11 (XLSX 29070 KB)

## Data Availability

The data analyzed during the study can be obtained from the corresponding author upon reasonable request.
