# Supplementary figures and images for: A novel prognostic model of methylation-associated genes in acute myeloid leukemia
Source: Clin Transl Oncol. 2023 Jan 30;25(6):1719–28. doi: 10.1007/s12094-022-03069-2 (PMC10203004; doi:10.1007/s12094-022-03069-2)

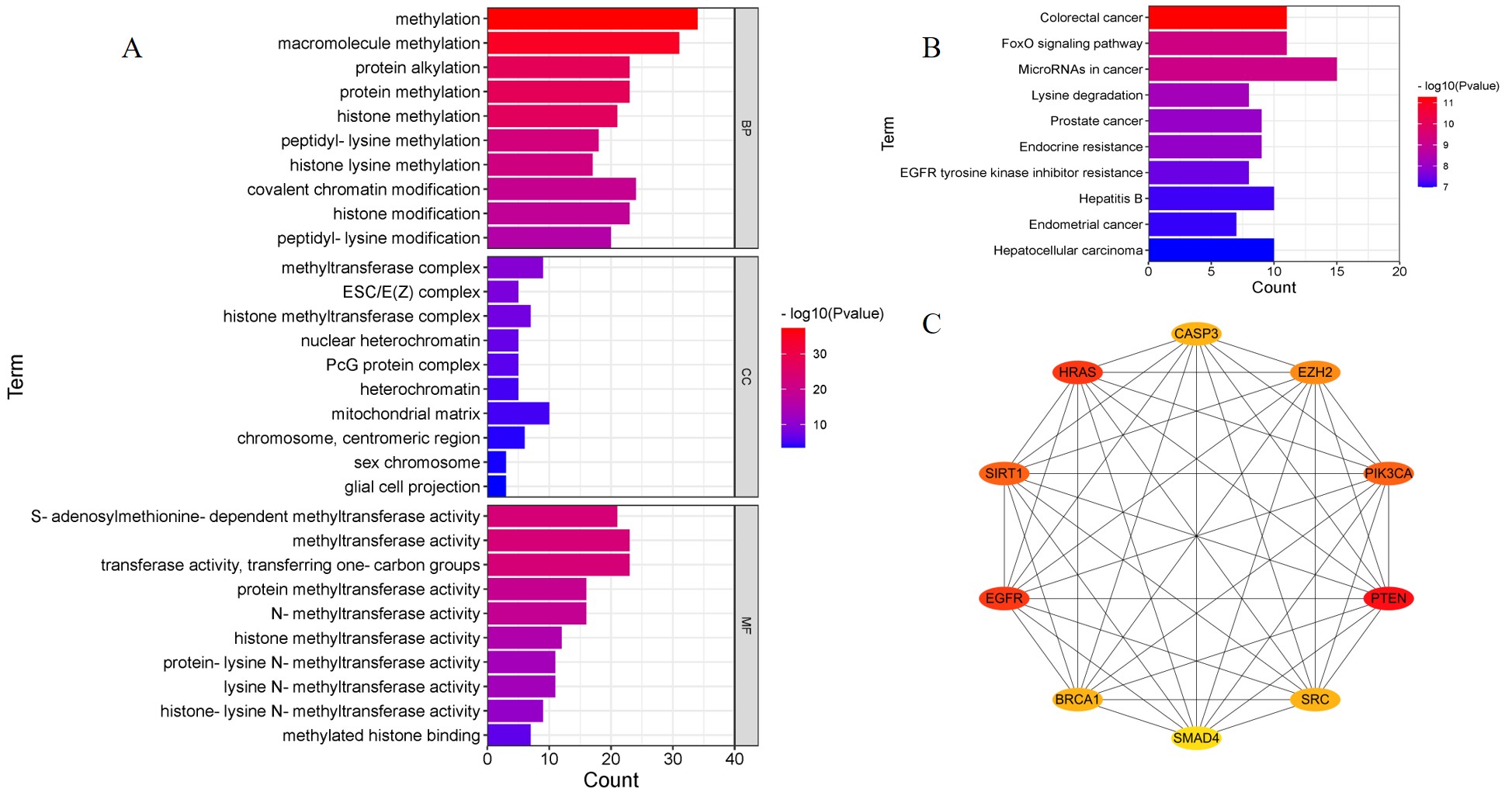

Supplement: Supplementary file 1 — Supplementary file1 Supplementary Fig. 1 The analysis of 76 prognostic MAGs. (A) The analysis of 76 prognostic MAGs based on GO enrichment analysis. (B) The analysis of 76 prognostic MAGs based on KEGG enrichment analysis. (C) Cluster analysis of interconnected genes by Cytoscape. (JPG 311 KB) [file 12094_2022_3069_MOESM1_ESM.jpg]

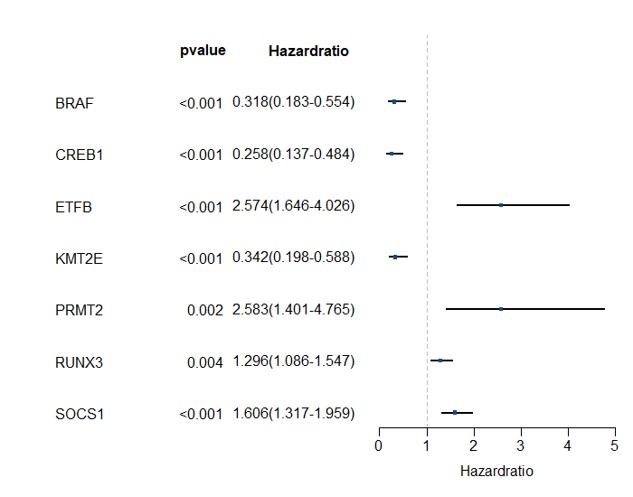

Supplement: Supplementary file 2 — Supplementary file2 Supplementary Fig. 2 Forest plot of univariate Cox regression analysis of 7 MRGs for overall survival in the training cohort. (JPG 34 KB) [file 12094_2022_3069_MOESM2_ESM.jpg]

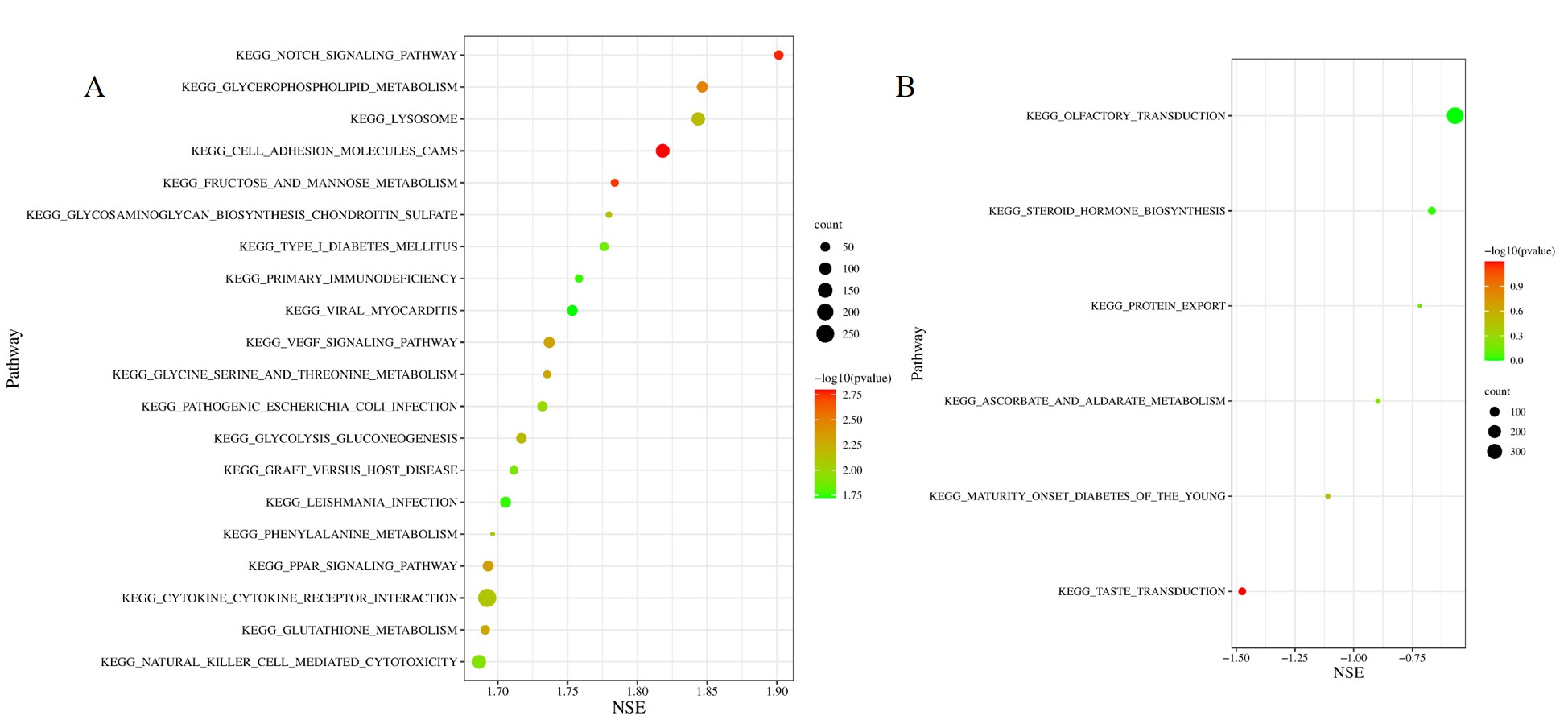

Supplement: Supplementary file 3 — Supplementary file3 Supplementary Fig. 3 (A) Significantly enriched pathways in the high-risk group in the training cohort. (B) Significantly enriched pathways in the low-risk group in the training cohort. (JPG 186 KB) [file 12094_2022_3069_MOESM3_ESM.jpg]

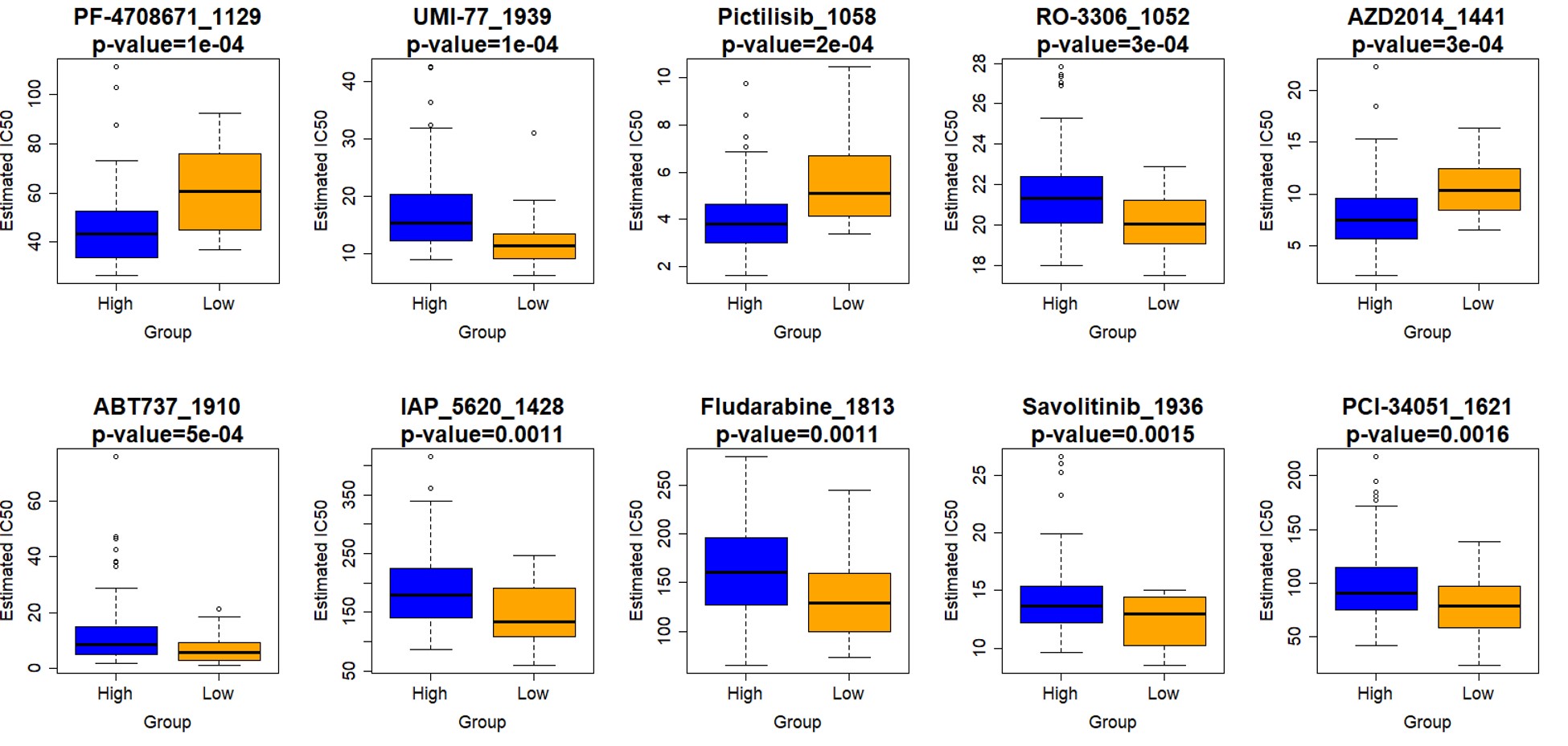

Supplement: Supplementary file 4 — Supplementary file4 Supplementary Fig. 4 The estimated IC50 of the top ten drugs with the most significant p values. Blue boxplots: IC50 values for the high-risk group; yellow boxplots: IC50 values for the low-risk group. (JPG 262 KB) [file 12094_2022_3069_MOESM4_ESM.jpg]

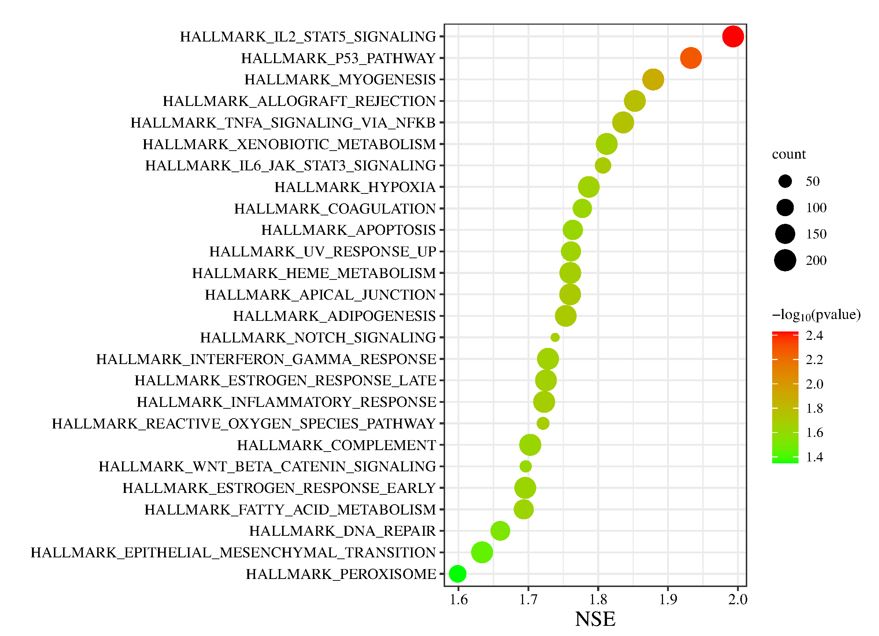

Supplement: Supplementary file 5 — Supplementary file5 (JPG 99 KB) [file 12094_2022_3069_MOESM5_ESM.jpg]
